# Ursodeoxycholic Acid Attenuates Lipopolysaccharide-Induced Myocardial Injury by Inhibiting Oxidative Stress, Inflammation, and Apoptosis: The Interplay of Sirt1/Nrf2 and Akt/NF-κB Signaling Pathways

**DOI:** 10.3390/ijms27062843

**Published:** 2026-03-20

**Authors:** Ranko Škrbić, Tatjana Milivojac, Milkica Grabež, Ljiljana Amidžić, Zorislava Bajic, Tanja Sobot, Nebojša Mandić-Kovačević, Snežana Uletilović, Đorđe Đukanović, Milica Gajic Bojic, Sanja Jovičić, Maja Barudžija, Nataša Vojinović, Miloš P. Stojiljković, Dragan M. Djuric, Hani Al-Salami, Sergey Bolevich, Momir Mikov

**Affiliations:** 1 Centre for Biomedical Research, Faculty of Medicine, University of Banja Luka, 78000 Banja Luka, The Republic of Srpska, Bosnia and Herzegovinaljiljana.amidzic@med.unibl.org (L.A.); nebojsa.mandic-kovacevic@med.unibl.org (N.M.-K.); snezana.uletilovic@med.unibl.org (S.U.); dragan.djuric@med.bg.ac.rs (D.M.D.);; 2 Department of Pharmacology, Toxicology and Clinical Pharmacology, Faculty of Medicine, University of Banja Luka, 78000 Banja Luka, The Republic of Srpska, Bosnia and Herzegovina; 3 Department of Pathophysiology, First Moscow State Medical University I.M. Sechenov, 119435 Moscow, Russia; 4 Academy of Science and Arts of the Republic of Srpska, 78000 Banja Luka, The Republic of Srpska, Bosnia and Herzegovina; 5 Department of Pathophysiology, Faculty of Medicine, University of Banja Luka, 78000 Banja Luka, The Republic of Srpska, Bosnia and Herzegovina; 6 Department of Hygiene, Faculty of Medicine, University of Banja Luka, 78000 Banja Luka, The Republic of Srpska, Bosnia and Herzegovina; 7 Department of Physiology, Faculty of Medicine, University of Banja Luka, 78000 Banja Luka, The Republic of Srpska, Bosnia and Herzegovina; 8 Department of Pharmacy, Faculty of Medicine, University of Banja Luka, 78000 Banja Luka, The Republic of Srpska, Bosnia and Herzegovina; 9 Department of Medical Biochemistry and Chemistry, Faculty of Medicine, University of Banja Luka, 78000 Banja Luka, The Republic of Srpska, Bosnia and Herzegovina; 10 Department of Histology and Embryology, Faculty of Medicine, University of Banja Luka, 78000 Banja Luka, The Republic of Srpska, Bosnia and Herzegovina; 11 Institute of Medical Physiology “Richard Burian”, Faculty of Medicine, University of Belgrade, 11000 Belgrade, Serbia; 12 The Biotechnology and Drug Development Research Laboratory, The School of Diagnostic and Therapeutic Sciences, and Curtin Medical Research Institute, Curtin University, Bentley, Perth, WA 6102, Australia; Hani.Al-Salami@curtin.edu.au; 13 Medical School, University of Western Australia, Perth, WA 6102, Australia; 14 Department of Pharmacology, Toxicology and Clinical Pharmacology, Faculty of Medicine, University of Novi Sad, 21000 Novi Sad, Serbia

**Keywords:** lipopolysaccharide, ursodeoxycholic acid, acute myocardial injury, Akt/NF-κB, SIRT1/Nrf2/HO-1, oxidative stress, apoptosis, cardioprotection

## Abstract

Oxidative stress is a critical pathophysiological factor in sepsis. Ursodeoxycholic acid (UDCA), a bile acid with anti-inflammatory, antioxidant, and anti-apoptotic properties, may protect against lipopolysaccharide (LPS)-induced myocardial injury. In an experimental study, 32 male Wistar rats were randomly assigned to four groups: control, LPS, UDCA, and UDCA + LPS. UDCA was administered orally for 10 days prior to LPS-induced endotoxemia. Serum levels of high-sensitive troponin I (hsTnI), homocysteine, and oxidative stress markers were measured, and immunohistochemistry and immunofluorescence were used to assess inflammation (nuclear factor kappa B, NF-κB), apoptosis (caspase 3), and signaling pathways related to protein kinase B (Akt)/NF-κB and silent information regulator 1 (SIRT1)/nuclear factor erythroid 2-related factor 2 (Nrf2)/heme oxygenase-1 (HO-1). UDCA pretreatment significantly reduced myocardial pathological changes, serum hsTnI, homocysteine, and total oxidative stress compared with LPS alone. It enhanced catalase (CAT) activity and glutathione (GSH) levels while lowering thiobarbituric acid reactive substances (TBARS) and nitrite concentrations in cardiac tissue. UDCA modulated cellular signaling by decreasing Akt phosphorylation and activating the SIRT1/Nrf2/HO-1 pathway. These results indicate that UDCA protects the heart from LPS-induced damage by reducing oxidative stress, inflammation, and apoptosis. UDCA modulates cellular signaling by decreasing pro-inflammatory pathways and activating anti-inflammatory pathways associated with SIRT1/Nrf2/HO-1 signaling, emphasizing its key role in myocardial protection during sepsis.

## 1. Introduction

Myocardial injury and cardiac dysfunction are frequent and severe complications of sepsis, strongly associated with increased mortality in septic patients [1,2], underscoring the urgent need for novel therapeutic approaches. Endotoxemia induces an acute systemic inflammatory response through activation of the innate immune system. Lipopolysaccharide (LPS), an endotoxin from Gram-negative bacteria, binds to Toll-like receptor 4 (TLR4), expressed on immune and non-immune cells. TLR4 activation triggers intracellular signaling cascades, among which nuclear factor kappa B (NF-κB) is a central transcription factor regulating inflammation, immune responses, and apoptosis [3,4]. NF-κB activation can suppress the antioxidant transcription factor nuclear factor erythroid 2-related factor 2 (Nrf2), reducing cellular antioxidant capacity and promoting oxidative stress. Conversely, anti-inflammatory interventions may activate Nrf2 and downstream effectors such as heme oxygenase-1 (HO-1), restoring redox balance [5,6].

Multiple upstream regulators converge on Nrf2/HO-1 signaling. Silent information regulator 1 (SIRT1), a NAD^+^-dependent deacetylase, modulates mitochondrial function and limits oxidative stress, inflammation, and apoptosis through Nrf2 and HO-1 activation [7,8]. Akt, also known as protein kinase B (PKB), is another key signaling molecule controlling cell survival, metabolism, and inflammatory responses. LPS potently activates PI3K/Akt signaling, which can regulate NF-κB activity while supporting adaptive responses to cellular stress [9,10,11,12,13,14]. This positions Akt as a central hub integrating pro-inflammatory and cytoprotective signals in sepsis-induced myocardial injury.

Bile acid signaling has emerged as an important modulator of cardiac protection. Agonists of the G-protein-coupled bile acid receptor 1 (GPBAR1/TGR5) and the farnesoid X receptor (FXR) exhibit antioxidant and anti-inflammatory effects in the heart [15,16,17]. Ursodeoxycholic acid (UDCA), a non-toxic hydrophilic bile acid, activates TGR5 and modulates FXR, enhancing Nrf2/HO-1-mediated antioxidant responses and inhibiting LPS-induced Akt/NF-κB signaling [18,19,20,21,22,23,24,25]. These effects collectively suggest that UDCA has potential as a cardioprotective agent under conditions of systemic inflammation and oxidative stress.

## 2. Results

All samples were collected 48 h after LPS injection, and this timing applies to all subsequent analyses described in Section 2.1, Section 2.2, Section 2.3, Section 2.4, Section 2.5 and Section 2.6.

### 2.1. Effects of UDCA Pretreatment on Biomarkers of LPS-Induced Acute Myocardial Injury

**Figure 1:**
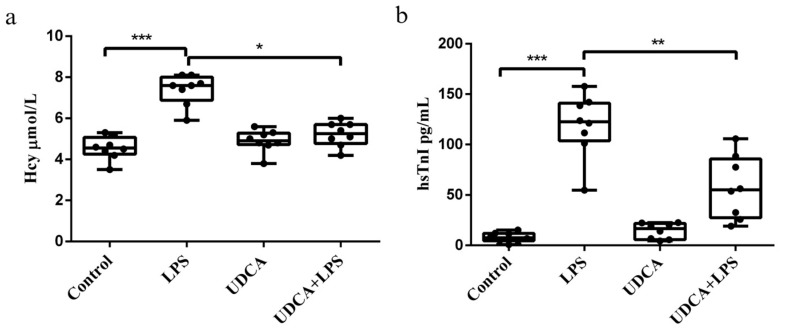
Effects of UDCA on Hcy and hsTnI level in LPS-induced acute myocardial injury. (**a**) Hcy level. (**b**) hsTnI level. Hcy—homocysteine; hsTnI—high-sensitivity troponin I. Hcy and hsTnI were measured in serum using commercially available assays on the Alinity c-analyzer (Abbott Laboratories, IL, USA). Data are expressed as mean ± SD (n = 8 per group). Control—group treated with UDCA solvent for 10 days p.o. and saline on day 10 i.p.; LPS—group treated with lipopolysaccharide 5.5 mg/kg i.p.; UDCA—group treated with ursodeoxycholic acid 25 mg/kg p.o. for 10 days; UDCA + LPS—group treated with ursodeoxycholic acid and lipopolysaccharide. One-way ANOVA, and Mann–Whitney tests were performed. *** *p* < 0.001, ** *p* < 0.01, * *p* < 0.05.

Homocysteine (Hcy) and high-sensitivity troponin I (hsTnI) were analyzed as biomarkers of LPS-induced acute myocardial injury. hsTnI is a specific marker of cardiomyocyte injury, whereas elevated Hcy levels are associated with oxidative stress and inflammatory processes contributing to myocardial damage. A significant increase in Hcy and hsTnI levels was observed in all rats treated with LPS (*p* < 0.001), while pretreatment with UDCA led to a significant reduction in Hcy and hsTnI levels (*p* < 0.05, *p* < 0.01, respectively). No changes in these parameters were induced by UDCA *per se* (Figure 1).

### 2.2. Effects of UDCA Pretreatment on Oxidative Stress Parameters in Serum and Heart Tissue Homogenate in LPS-Induced Acute Myocardial Injury

**Figure 2:**
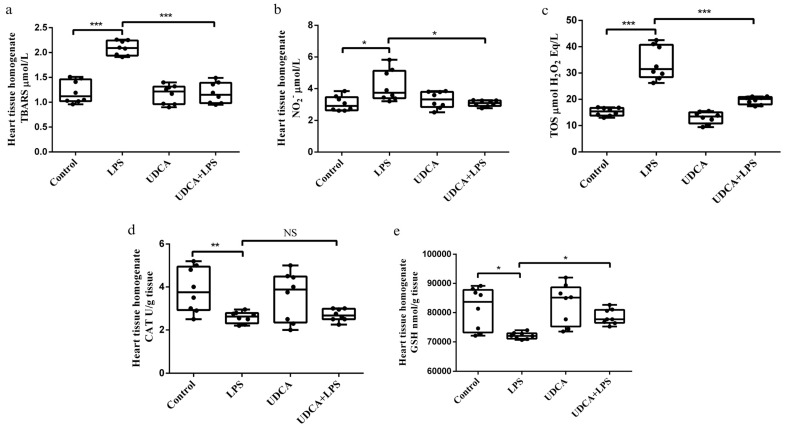
Effects of UDCA on oxidative stress markers in heart tissue homogenate. (**a**) TBARS level; (**b**) NO_2_^−^ level; (**c**) TOS level; (**d**) CAT activity; (**e**) GSH level. TBARS—thiobarbituric acid reactive substances; NO_2_^−^—nitrites; TOS—total oxidative status; CAT—catalase; GSH—glutathione. Oxidative stress markers were measured in heart tissue homogenates using the following methods: (**a**) TBARS—determined spectrophotometrically based on the reaction of thiobarbituric acid with malondialdehyde; (**b**) NO_2_^−^—determined as nitrites using the Griess method; (**c**) TOS—measured by the total oxidants in the sample oxidizing ferrous ions to ferric ions, which then react with xylenol orange; (**d**) CAT activity—assessed spectrophotometrically following the Beutler method; (**e**) GSH—measured spectrophotometrically using the Beutler method. Data are expressed as mean ± SD (n = 8 per group). Control—group treated with UDCA solvent for 10 days p.o. and saline on day 10 i.p.; LPS—group treated with lipopolysaccharide 5.5 mg/kg i.p.; UDCA—group treated with ursodeoxycholic acid 25 mg/kg p.o. for 10 days; UDCA + LPS—group treated with ursodeoxycholic acid and lipopolysaccharide. One-way ANOVA, and Mann–Whitney test were performed. *** *p* < 0.001, ** *p* < 0.01, * *p* < 0.05, NS—not significant.

In LPS-induced acute myocardial injury, UDCA showed antioxidant effects (Figure 2). ROS plays a key role in oxidative stress, and prooxidative-marker total oxidative status (TOS) in serum was analyzed as a representative of ROS. In heart homogenate, thiobarbituric acid reactive substances (TBARS), a marker of lipid peroxidation, showed a significant increase in the LPS-treated group compared with the control group (*p* < 0.001). The application of UDCA significantly reduced TBARS levels (*p* < 0.001) (Figure 2a). LPS caused an increase in NО_2_^−^ levels (*p* < 0.05) compared with the control, and the application of UDCA significantly attenuated this effect (*p* < 0.05) (Figure 2b). TOS levels were significantly increased in the LPS group compared with the control group (*p* < 0.001), indicating the involvement of LPS in oxidative stress in acute heart injury. UDCA administration significantly lowered TOS levels in LPS-treated rats but not in control ones (*p* < 0.001) (Figure 2c). Catalase (CAT) activity and glutathione (GSH) level were examined as components of the antioxidant defense system. CAT activity was decreased in the LPS-treated group (*p* < 0.05) compared with the control group, while pretreatment with UDCA partially restored CAT activity, although the difference between the LPS and UDCA + LPS groups did not reach statistical significance. GSH levels were significantly decreased in the LPS group (*p* < 0.05), and UDCA pretreatment significantly increased GSH levels compared with LPS alone (*p* < 0.05) (Figure 2d,e).

### 2.3. Effects of UDCA Pretreatment on Histopathological Characteristics of the Heart in LPS-Induced Acute Myocardial Injury

**Figure 3:**
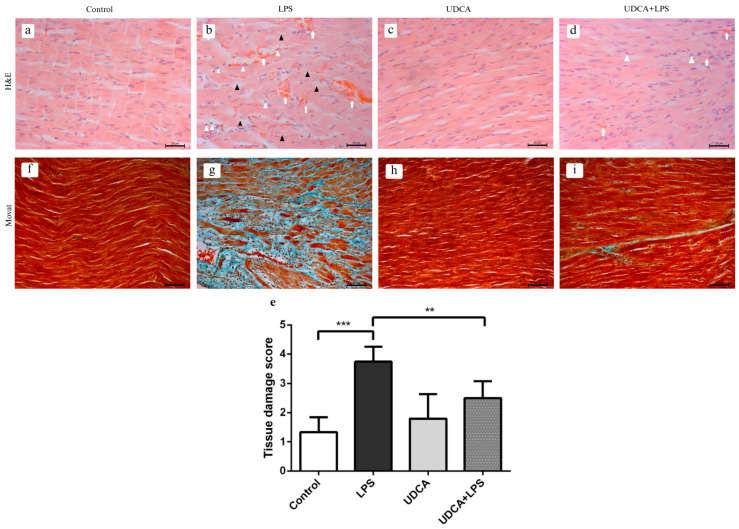
Effects of UDCA on LPS-induced myocardial injury and interstitial fibrosis in rat heart tissue. Heart tissue sections were analyzed by H&E and Movat pentachrome staining to evaluate the severity of myocardial damage and interstitial changes. Control—group treated with UDCA solvent for 10 days p.o. and saline on day 10 i.p.; LPS—group treated with lipopolysaccharide 5.5 mg/kg i.p.; UDCA—group treated with ursodeoxycholic acid 25 mg/kg p.o. for 10 days; UDCA + LPS—group treated with ursodeoxycholic acid and lipopolysaccharide. Panels (**a**–**d**): H&E staining, magnification 400×, scale bar: 25 µm. (**e**) tissue damage score. Panels (**f**–**i**): Movat pentachrome staining, magnification 200×, scale bar: 50 µm. Data are expressed as mean ± SD (n = 8 per group). One-way ANOVA and Mann–Whitney tests were performed. *** *p* < 0.001, ** *p* < 0.01.

H&E-stained myocardial sections were analyzed to assess the severity of cardiac tis-sue damage. In the control group (Figure 3a), the myocardium displayed normal architecture, with preserved cardiomyocyte branching, normal nuclei, and clearly visible intercalated discs. In the LPS group (Figure 3b), significant degeneration and vacuolization of cardiomyocytes were observed (black arrowheads), accompanied by interstitial hemorrhage, edema (white arrows), and infiltration of inflammatory cells (white arrowheads), indicating marked myocardial injury. The UDCA group (Figure 3c) showed mostly preserved myocardial architecture, with cardiomyocytes exhibiting normal nuclei. In the UDCA + LPS group (Figure 3d), cardiomyocyte morphology was largely maintained, with visible intercalated discs (black arrowheads), only discrete interstitial hemorrhage (white arrows), and minimal inflammatory cell infiltration (white arrowheads), reflecting attenuation of LPS-induced damage. Semi-quantitative scoring confirmed these observations, with a significant increase in myocardial damage in the LPS group compared with control (*p* < 0.001) and partial restoration in the UDCA + LPS group (Figure 3e).

Movat pentachrome staining demonstrated normal histology in the control group, with red-stained cardiomyocytes and clearly visible endomysium. In the LPS group, inflammatory infiltrates, early signs of fibrosis, mucopolysaccharide accumulation (blue staining), and damaged cardiomyocytes were observed. The UDCA group resembled the control group, while the UDCA + LPS group showed mostly preserved myocardium, although scattered inflammatory cells and slightly enlarged cardiomyocytes were present (Figure 3f–i).

### 2.4. Effects of UDCA Pretreatment on pAkt Activation in LPS-Induced Acute Myocardial Injury

**Figure 4:**
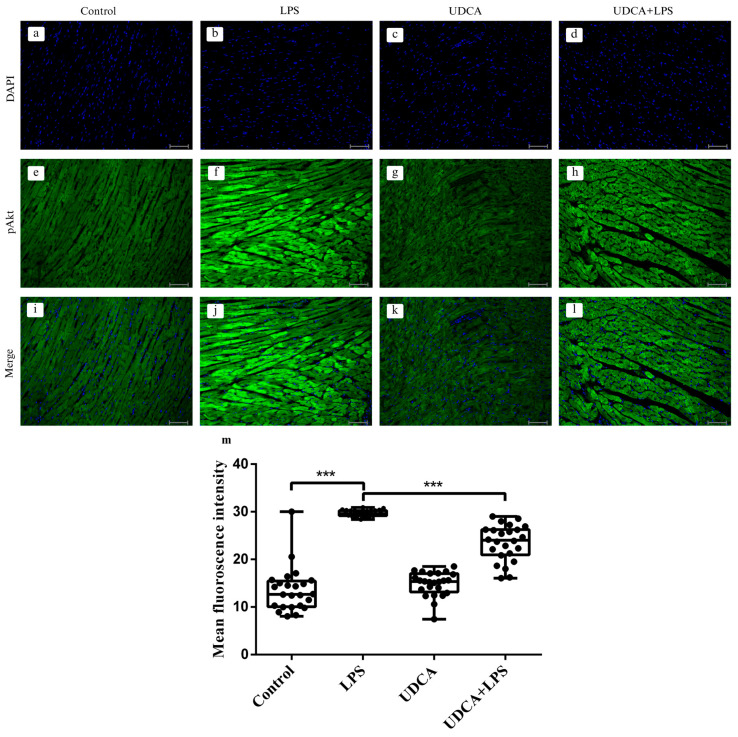
Immunofluorescence analysis of phosphorylated Akt (pAkt) in rat myocardium (magnification 200×, scale bar 50 μm). Heart tissue sections were stained for pAkt using specific primary antibodies and Alexa Fluor^®^ 488-conjugated secondary antibodies. Nuclei were counterstained with DAPI. Control—group treated with UDCA solvent for 10 days p.o. and saline on day 10 i.p.; LPS—group treated with lipopolysaccharide 5.5 mg/kg i.p.; UDCA—group treated with ursodeoxycholic acid 25 mg/kg p.o. for 10 days; UDCA + LPS—group treated with ursodeoxycholic acid and lipopolysaccharide. Panels (**a**–**d**): DAPI nuclear staining (blue); panels (**e**–**h**): pAkt fluorescence (green); panels (**i**–**l**): merged images; panel (**m**): quantification of mean fluorescence intensity of pAkt. Data are expressed as mean ± SD (n = 8 per group). One-way ANOVA, Bonferroni, and Mann–Whitney tests were performed. *** *p* < 0.001.

Immunofluorescence analysis of phosphorylated Akt (pAkt) revealed significant differences among the experimental groups (Figure 4). In the control group, a low basal level of pAkt immunoreactivity with a homogeneous tissue distribution was observed (Figure 4a,e). DAPI staining (Panels a–d) showed similar distribution and density of nuclei among groups, indicating that the observed differences in pAkt signal were not due to differences in cell number. LPS treatment led to a marked increase in pAkt fluorescence compared with the control (*p* < 0.001) (Figure 4b,f,m), indicating a significant increase in Akt phosphorylation in the myocardium compared with the control group. Administration of UDCA alone did not significantly affect pAkt expression compared with the control group (Figure 4c,g). However, UDCA pretreatment significantly reduced the pAkt fluorescence intensity compared with the LPS group (*p* < 0.001), indicating that UDCA treatment effectively attenuates LPS-induced Akt phosphorylation (Figure 4d,h,m), although the signal remained higher than in the control group. This finding suggests that UDCA partially attenuates LPS-induced Akt hyperactivation, indicating a modulatory rather than a fully suppressive effect on this signaling pathway.

### 2.5. Effects of UDCA Pretreatment on NF-κB and Caspase 3 in LPS-Induced Acute Myocardial Injury

**Figure 5:**
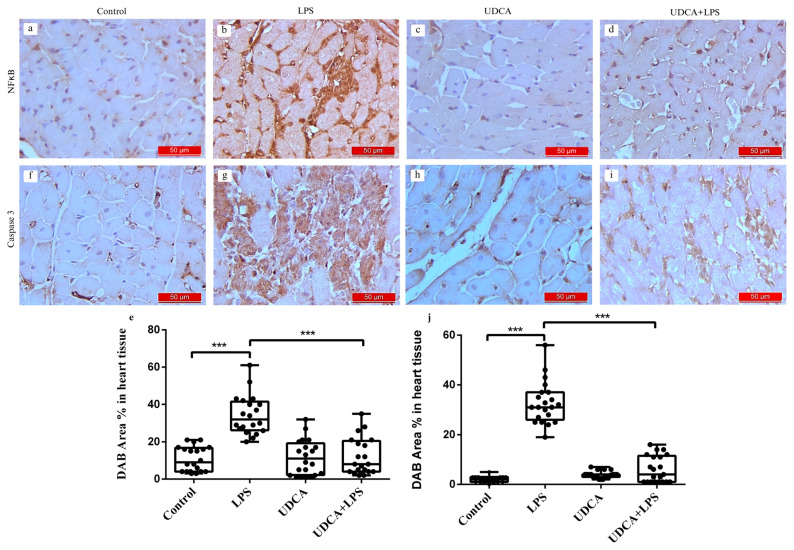
Immunohistochemical staining and quantitative analysis of NF-κB and caspase 3 expression in the myocardium of control, LPS, UDCA and UDCA + LPS rats. Panels a–d show representative immunohistochemical images of NF-κB expression in the myocardium: (**a**) control, (**b**) LPS, (**c**) UDCA, and (**d**) UDCA + LPS rats. Panel (**e**) displays the quantification of DAB Area % in heart tissue of NF-κB. Panels (**f**–**i**) show representative images of caspase 3 expression in the myocardium: (**f**) control, (**g**) LPS, (**h**) UDCA, and (**i**) UDCA + LPS rats. Panel (**j**) displays the quantification of DAB Area % in heart tissue of caspase 3. Data are expressed as mean ± SD. Control—group treated with UDCA solvent for 10 days p.o. and saline on day 10 i.p.; LPS—group treated with lipopolysaccharide 5.5 mg/kg i.p.; UDCA—group treated with ursodeoxycholic acid 25 mg/kg p.o. for 10 days; UDCA + LPS—group treated with ursodeoxycholic acid and lipopolysaccharide. One-way ANOVA, Bonferroni test, and Mann–Whitney test were performed. *** *p* < 0.001.

Immunohistochemical assessment and chromogen 3,3′-diaminobenzidine tetrahydrochloride (DAB) Area % in heart tissue of NF-κB and caspase 3, Figure 5 showed a significant increase in NF-κB and pro-apoptotic marker caspase 3 immunoreactivity in myocardial tissue in LPS group compared with the control group (*p* < 0.001) (Figure 5b,g,e,j). Rats pretreated with UDCA and injected with LPS exhibited a marked reduction in the intensity of NF-κB and caspase 3 immunostaining (*p* < 0.001) (Figure 5d,i,e,j). Rats that received only UDCA (Figure 5c,h), similar to the control group (Figure 5a,f), showed no detectable presence of NF-κB and caspase 3 in the immunohistochemical analyses. % Area DAB represents the proportion of myocardial tissue positively stained for the target antigen and was used to quantify NF-κB and caspase-3 expression.

### 2.6. Effects of UDCA Pretreatment on SIRT1, Nrf2, and HO-1 in LPS-Induced Acute Myocardial Injury

**Figure 6:**
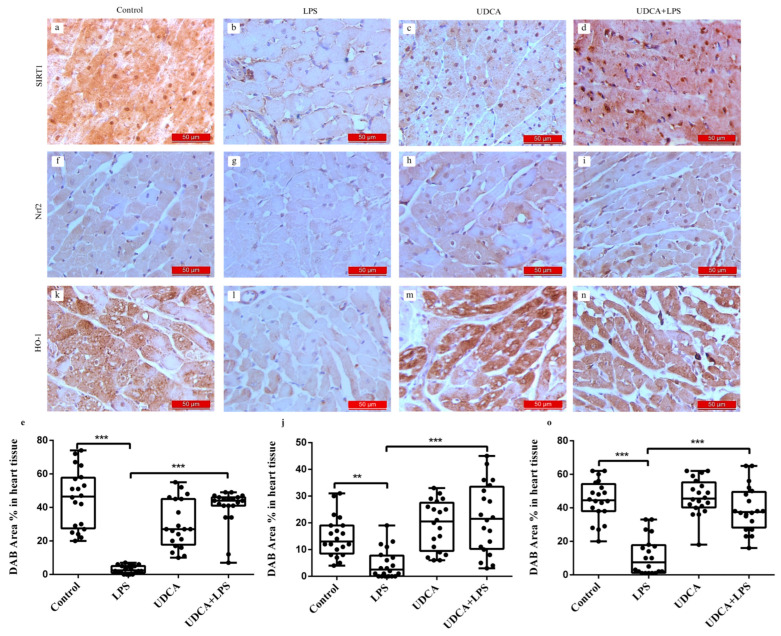
Immunohistochemical staining and quantitative analysis of SIRT1, Nrf2 and HO-1 expression in the myocardium of control, LPS, UDCA and UDCA + LPS rats. Panels (**a**–**d**) show representative immunohistochemical images of SIRT1 expression in the myocardium: (**a**) control, (**b**) LPS, (**c**) UDCA, and (**d**) UDCA + LPS rats. Panel (**e**) displays the quantification of DAB Area % in heart tissue of SIRT1. Panels (**f**–**i**) show representative images of Nrf2 expression in the myocardium: (**f**) control, (**g**) LPS, (**h**) UDCA, and (**i**) UDCA + LPS rats. Panel (**j**) displays the quantification of DAB Area % in heart tissue of Nrf2. Panels (**k**–**n**) show representative immunohistochemical images of HO-1 expression in the myocardium: (**k**) control, (**l**) LPS, (**m**) UDCA, and (**n**) UDCA + LPS rats. Panel (**o**) displays the quantification of DAB Area % in heart tissue of HO-1. Data are expressed as mean ± SD. Control—group treated with UDCA solvent for 10 days p.o. and saline on day 10 i.p.; LPS—group treated with lipopolysaccharide 5.5 mg/kg i.p.; UDCA—group treated with ursodeoxycholic acid 25 mg/kg p.o. for 10 days; UDCA + LPS—group treated with ursodeoxycholic acid and lipopolysaccharide. The brown color indicates NF-κB, caspase 3, SIRT1, Nrf2, and HO-1 positivity. One-way ANOVA, Bonferroni test, and Mann–Whitney test were performed. ** *p* < 0.01, *** *p* < 0.001.

Immunohistochemical analysis and DAB staining Area % in heart tissue of SIRT1, Nrf2, and HO-1 revealed a significant decrease in the immunoreactivity of these markers in myocardial tissue from the LPS-treated group (Figure 6b,g,l). In contrast, rats pretreated with UDCA and injected with LPS showed an increase in immunoreactivity in SIRT1, Nrf2, and HO-1 immunostaining (Figure 6d,i,n). Similar to the control group (Figure 6a,f,k), rats treated with UDCA alone exhibited positive immunohistochemical findings for SIRT1, Nrf2, and HO-1 (Figure 6c,h,m). The LPS group displayed the lowest DAB Area % in heart tissue for SIRT1, Nrf2, and HO-1 (*p* < 0.001, *p* < 0.01, and *p* < 0.001, respectively) (Figure 6e,j,o). % Area DAB represents the proportion of myocardial tissue positively stained for the target antigen and was used to quantify SIRT1, Nrf2 and HO-1 expression.

## 3. Discussion

LPS induces acute systemic inflammation by activating the immune system through TLR4 [3], and excessive TLR4 activation can lead to systemic inflammatory response syndrome (SIRS) [26]. Studies in humans and rodents suggest that SIRS is associated with redox imbalance. Increased ROS production and impaired antioxidant defenses can cause mitochondrial dysfunction; disrupt cellular homeostasis; and induce oxidative damage to DNA, proteins, and lipids, leading to tissue injury and apoptosis [1]. The heart is particularly vulnerable to sepsis-induced damage, with myocardial dysfunction resulting from ROS, inflammation, and microcirculatory disturbances being a severe complication [27]. Cardiovascular manifestations of sepsis include shock, pericardial effusion, and ECG changes that may mimic acute myocardial infarction [28], along with biochemical markers of injury such as hsTnI and Hcy.

Our study showed a significant increase in hsTnI and Hcy levels in LPS-treated rats, indicating severe myocardial injury under endotoxemia. Elevated TnI specifically reflects cardiac myocyte necrosis [29,30] and has been linked to increased mortality in septic patients, highlighting its diagnostic value [31,32]. UDCA administration attenuated the LPS-induced hsTnI elevation, suggesting a cardioprotective effect. Similarly, a recent study from our laboratory demonstrated that UDCA pretreatment significantly reduces serum hsTnI in isoprenaline-induced myocardial injury in rats [33].

Hcy is closely linked to sepsis through mechanisms involving oxidative stress, inflammation, and endothelial dysfunction [34]. Peripheral blood mononuclear cells can produce Hcy, suggesting that immune activation during sepsis contributes to hyperhomocysteinemia and worsens disease progression [35]. Hcy acts as a pro-inflammatory mediator, stimulating cytokine synthesis and promoting oxidative stress, which is associated with increased mortality in sepsis [36,37]. UDCA can modulate Hcy levels associated with its antioxidant effects, inhibiting lipid peroxidation and ROS-induced oxidative stress through the PI3K/Akt/Nrf2 pathway [38]. In our study, LPS elevated Hcy levels, which were reduced by UDCA pretreatment, indicating its potential to regulate metabolic responses during inflammation.

Oxidative stress, marked by increased ROS and reduced antioxidant enzyme levels, is a key mechanism in LPS-induced endotoxemia and myocardial injury [30]. Excess ROS can activate NF-κB, promoting inflammation, apoptosis, and cardiac dysfunction [39]. UDCA mitigates oxidative stress by scavenging ROS and enhancing endogenous antioxidant defenses [40]. In this study, LPS increased TBARS and nitrites (NO_2_^−^) in cardiac tissue and TOS in serum, while reducing CAT activity and GSH levels in the cardiac homogenate. UDCA pretreatment reversed these changes, significantly decreasing TBARS, NO_2_^−^, and TOS and restoring CAT activity and GSH, confirming its antioxidant effects in LPS-induced myocardial damage.

The results of the present in vivo study clearly demonstrate that LPS administration leads to a significant activation of the Akt signaling pathway, thereby confirming its involvement in the cellular response to an inflammatory stimulus. The obtained results are fully consistent with previously published data showing that LPS is a potent activator of the PI3K/Akt signaling pathway in various cell types, where PI3K/Akt pathway activation is involved in the regulation of pro-inflammatory cytokine production and NF-κB activation [12,13,41,42,43]. This confirms the central role of the Akt signaling pathway in transmitting the inflammatory signal to downstream effectors in the pathophysiology of LPS-induced inflammation. In contrast, treatment with UDCA exerts a pronounced protective and modulatory effect, as reflected by the attenuation of excessive LPS-induced Akt phosphorylation. These findings suggest that UDCA may alleviate inflammation-mediated dysregulation of intracellular signaling and contribute to the maintenance of cellular homeostasis. Such effects indicate the potential cytoprotective and anti-inflammatory properties of UDCA, which is consistent with the findings of Li X et al. [25], who demonstrated that UDCA derivatives markedly inhibit the pro-inflammatory signaling pathways, including the Akt/NF-κB axis, thereby reducing the LPS-induced inflammatory response.

The results of the present study clearly showed the reduction in the expression of NF-κB, a key factor in inflammatory responses, and caspase 3, a major executor of apoptosis, in the myocardium of LPS-treated rats, which further confirms the protective role of UDCA. NF-κB is associated with promoting inflammation and can contribute to myocardial injury due to excessive activation of the inflammatory response [44]. The reduction in NF-κB expression mediated by UDCA suggests the capacity of this bile acid to inhibit LPS-induced inflammatory processes, potentially through the upregulation of Nrf2 [45]. UDCA demonstrates the ability to stabilize membranes and prevent apoptosis [46]. Reduction in caspase 3 expression indicates decreased apoptosis in tissues treated with UDCA, which is crucial for protecting myocardial cells from programmed death due to endotoxin-induced damage.

Nrf2 plays a key role in maintaining redox homeostasis and cytoprotection and is a crucial regulator of cellular resistance to oxidative stress. Its activation is associated with the induction of superoxide and peroxide catabolism through the expression of antioxidant genes and the promotion of the synthesis of antioxidant proteins and enzymes such as GSH, SOD, CAT, and HO-1 [47]. In addition to the classical activation pathway, Nrf2 can also be regulated through the protein p62, which acts as an autophagy receptor responsible for the degradation of proteins and mitochondria. The p62-mediated Nrf2 activation leads to the increased expression of oxidoreductase (NQO1), GSTs, and anti-apoptotic proteins like Bcl-2 and Bcl-xL, thereby reducing ROS levels and protecting cells from oxidative stress [48].

Studies have confirmed that SIRT1 facilitates the activation and translocation of Nrf2 into the nucleus, enhancing its DNA-binding activity and transcriptional function. This, in turn, prevents oxidative tissue damage and apoptosis induced by oxidative stress [47]. Increased Nrf2 expression boosts the levels of HO-1, which further promotes the degradation of IκBα, thereby limiting NF-κB activation and mitigating the inflammatory response [49]. Numerous studies suggest that HO-1 represents one of the central points of interaction between the Nrf2 and NF-κB pathways. HO-1 can mediate inflammatory processes by inhibiting the nuclear translocation of the NF-κB pathway through its end products, like carbon monoxide and bilirubin, both of which have been shown to suppress NF-κB activation [49,50].

The results of this study demonstrated that UDCA pretreatment significantly increased the expression of SIRT1, Nrf2, and HO-1 in the myocardium of LPS-treated rats, indicating the activation of protective mechanisms within cardiomyocytes, which aligns with other studies that have shown that the mechanism of the cytoprotective action of UDCA is related to the activation of the Nrf2 signaling pathway [51]. The increased expression of SIRT1 and Nrf2, along with the increase in HO-1, suggests that UDCA enhances the antioxidant defense of the myocardium by activating the SIRT1/Nrf2/HO-1 signaling pathway. In addition to the classical activation pathway, emerging evidence indicates that the NAD-dependent deacetylase Sirt1 can promote the stabilization and activation of Nrf2 by reducing its acetylation, thus enhancing its transcriptional activity and downstream antioxidant effects. Sirt1-mediated modulation of the Keap1/Nrf2 pathway has been associated with decreased oxidative stress, suppressed inflammation, and reduced apoptosis in multiple experimental models [52]. While the present study did not directly assess Nrf2 acetylation or Sirt1-dependent deacetylation of Nrf2 or Keap1, the importance of this axis in orchestrating cellular antioxidant responses suggests that UDCA’s protective effects may also involve enhancement of intrinsic antioxidative signaling through Sirt1/Nrf2 regulation. Although UDCA treatment was associated with modulation of the SIRT1/Nrf2/HO-1 and Akt/NF-κB pathways, causal relationships were not directly tested. Further mechanistic studies using pathway-specific inhibitors or genetic approaches are needed to clarify these interactions in sepsis-induced myocardial injury.

However, since this study evaluated only preventive administration prior to LPS challenge, further research is needed to assess the therapeutic efficacy of UDCA after the onset of sepsis. A limitation of the present study is that cardiac function was not directly measured; although histological changes, together with biochemical and oxidative markers, clearly indicate myocardial damage, functional tests would be required to determine the actual impact on heart performance. Additionally, phosphorylation was assessed only by immunofluorescence, without complementary biochemical validation (e.g., Western blot), representing a further methodological limitation. Finally, the relatively small sample size and exploratory nature of the study should be considered when interpreting the results, and future studies with larger cohorts are warranted to confirm these findings. Nevertheless, UDCA (25 mg/kg orally for 10 days) and LPS (5.5 mg/kg i.p.) were chosen based on prior rat studies [18], providing a translationally relevant model to explore mechanisms that may inform future human research.

In conclusion, UDCA exhibits significant cardioprotective potential under conditions of LPS-induced endotoxemia. This effect is associated with modulation of the Akt signaling pathway, whereby its pathological hyperactivation is prevented while its pro-survival function is preserved. In parallel, activation of the SIRT1/Nrf2/HO-1 signaling axis contributes to the enhancement of antioxidant defense, suppression of inflammatory and pro-apoptotic processes, and stabilization of cellular homeostasis. The integration of these mechanisms indicates that UDCA does not act as a non-selective inhibitor of intracellular signaling pathways but rather as a regulatory agent that restores the balance between inflammation, oxidative stress, and cell survival, thereby confirming its therapeutic potential in protecting the myocardium during systemic inflammation.

## 4. Materials and Methods

### 4.1. Animals

Male Wistar albino rats (n = 32), weighing 200–250 g, were housed in cages under standardized laboratory conditions (temperature 21 ± 2 °C, 12 h light/dark cycle, humidity 55 ± 5%), with unlimited access to food and water. The experimental procedures, protocols, and animals were approved by the Ethics Committee for Welfare of Experimental Animals of the Faculty of Medicine, University of Banja Luka (approval No. 18/1.190-1/22, dated 9 March 2022). All experiments were conducted following the relevant regulations (EU Directive for the protection of vertebrates used for experimental and scientific purposes 86/609/EEC), with strict adherence to ethical principles.

### 4.2. Experimental Protocol

The rat endotoxemia model is based on the intraperitoneal administration of a non-lethal single dose of *Escherichia coli* lipopolysaccharide (0.25 LD50/kg) at a dose of 5.5 mg/kg BW; a model that has shown the strongest inflammatory effects in various animal models of sepsis [4]. The doses of UDCA (25 mg/kg, orally for 10 days) and LPS (5.5 mg/kg, i.p.) were selected based on previous rat studies demonstrating anti-inflammatory effects of UDCA and consistent induction of systemic inflammation by LPS [18]. After a 1-week adaptation period, the rats were randomly assigned to four experimental groups, although no formal randomization software or sequence was used. All researchers performing the assays and image analyses were blinded to the group assignments. The groups were as follows: (a) control group (n = 8), which received a bile acid vehicle (propylene glycol) orally for 10 days and saline intraperitoneally on the tenth day; (b) LPS group (n = 8), treated identically to the control group, except that on the tenth day, *Escherichia coli* endotoxin was administered intraperitoneally instead of saline; (c) UDCA group (n = 8), which received UDCA 25 mg/kg BW orally for 10 days and saline intraperitoneally on the tenth day; (d) LPS + UDCA group (n = 8), treated identically to the UDCA group, but with the administration of *Escherichia coli* endotoxin intraperitoneally on the tenth day.

### 4.3. Blood and Tissue Collection, Heart Tissue Homogenization

Forty-eight hours after LPS injection, the rats were anaesthetized with a combination of ketamine (90 mg/kg) and xylazine (10 mg/kg). Blood samples were collected from the aorta; the serum was separated from whole blood and stored at −80 °C until analysis. The hearts were isolated, with one half placed in plastic containers with 10% formalin for immunohistochemical, immunofluorescent and histological analyses, while the other half was rinsed with cold saline solution and then frozen at −20 °C until homogenate preparation. The tissue homogenate was prepared in cold phosphate buffer (pH 7.4) using an HG-15D homogenizer (Witeg Labortechnik GmbH, Wertheim, Germany) and centrifuged at +4 °C and 1200× *g*.

The serum was used to evaluate the levels of hsTnI and Hcy and the total oxidative status (TOS). In the heart tissue homogenate supernatant, oxidative stress markers were assessed, including levels of thiobarbituric acid reactive substances (TBARS), nitric oxide (NO) measured as nitrites (NO_2_^−^), glutathione (GSH), and the activities of catalase (CAT).

The concentration of Hcy and hsTnI were measured by methods developed by Abbot Diagnostics on the Alinity c-analyzer (Abbott Laboratories, Chicago, IL, USA) [53].

The principle of the method for TOS determination is based on the fact that total oxidants in the sample oxidize ferrous ions to ferric ions, which then react with xylene orange in an acidic medium to form a colored complex measurable spectrophotometrically at a wavelength of 560 nm [54]. Measurements were performed on an ILAB 300+ analyser (Instrumentation Laboratory, Bedford, MA, USA) using a 546 nm filter.

The level of TBARS was determined spectrophotometrically using a Shimadzu UV 1800 device (Shimadzu Corporation, Kyoto, Japan), based on the reaction between thiobarbituric acid (TBA) and malondialdehyde (MDA).

The NO, determined in heart homogenate, is unstable and rapidly converts into derivatives such as nitrites, which can be quantified using the Griess method. This method utilizes 30% sulfosalicylic acid and Griess reagent, with measurements conducted at a wavelength of 550 nm [55].

The activity of CAT, as well as the levels of GSH in heart tissue homogenate, were analyzed spectrophotometrically following the Beutler method [56,57,58].

### 4.4. Histopathological Analysis

The heart samples were fixed in 4% formaldehyde for 48 h and processed using a Leica TP 1020 tissue processor (Leica Biosystems, Wetzlar, Germany). They were then embedded in paraffin blocks, sectioned using a Rotatory 3003 pfm microtome (Pfm Medical Gmbh, Cologne, Germany) at a thickness of 5 μm, and stained in two ways, with hematoxylin and eosin (H&E) and with Movat pentachrome staining. Examination was performed on a binocular Leica DM 6000 microscope (Leica Microsystems, Wetzlar, Germany), equipped with a Leica DFC310FX camera (Leica Microsystems, Wetzlar, Germany).

Rat heart sections stained with (H&E) from each group were examined morphologically and scored according to Bajic et al. [59]: Score 1—normal findings without morphological changes; Score 2—single cell with granules of small size slightly expanded and normal nuclei; Score 3—more than 50% cells with mild cytoplasmic vacuolization and nucleoplasm and pyknotic nuclei, indicating overall damage; Score 4—all cells with pronounced vacuolization of cytoplasm and nucleoplasm and pyknotic nuclei indicating moderate damage; Score 5—pronounced plasmolysis and karyolysis and diffuse infiltration of polymorphonuclear cells that surround phagocyte dead cells, bleeding, and interstitial oedema, suggesting severe damage.

Movat staining was performed using the Abcam Movat Pentachrome Stain Kit (ab245884, Modified Russell–Movat, Cambridge, UK), following the manufacturer’s instructions in 23 steps. Staining interpretation: elastic fibers—black to blue/black; nuclei—blue/black; collagen—yellow to red; reticular fibers—yellow; mucin—bright blue; fibrin—bright red; muscle—red.

### 4.5. Immunohistochemical Analysis

Antigen retrieval was performed using a citrate buffer (pH 6.0) in PT module (Pretreatment Module^TM^, Lab Vision Corporation, Fremont, CA, USA)*,* at 95 °C for 20 min. After cooling to room temperature, primary antibodies were applied to tissue sections and incubated overnight at 4 °C in a humid chamber. Detection was performed using secondary antibodies conjugated with horseradish peroxidase (HRP) and a polyvalent detection system (UltraVision Detection System HRP Polymer and DAB Plus Chromogen, Thermo Fisher Scientific, Waltham, MA, USA). Immunoreactivity was visualized using 3,3′-diaminobenzidine tetrahydrochloride (DAB), followed by hematoxylin and eosin (H&E) counterstaining.

The primary antibodies used were as follows:

SIRT1: rabbit polyclonal antibody (Invitrogen, PA5-116530, Waltham, MA, USA; dilution 1:300).

Nrf2: rabbit polyclonal antibody (Abcam, ab31163, Cambridge, UK; dilution 1:100).

HO-1: rabbit polyclonal antibody (Invitrogen, PA5-77833, Waltham, MA, USA; dilution 1:500).

NF-κB p65: mouse monoclonal IgG1 antibody (Santa Cruz Biotechnology, sc-514451, Dallas, TX, USA; dilution 1:100).

Caspase-3: rabbit monoclonal antibody (Cell Signaling Technology, #9664, Danvers, MA, USA; dilution 1:400).

The samples were examined using a Leica DM2500 optical microscope (Leica Microsystems, Wetzlar, Germany) and images were captured with an MC170HD camera (Leica Microsystems, Wetzlar, Germany) at 400× magnification and archived in TIFF format (2592 × 1944 pixels). Morphometric and photometric analysis was performed using Fiji software (National Institutes of Health, Bethesda, MD, USA; version 1.54f). The DAB fraction area in myocardial tissue (% Area DAB) was calculated as

% Area DAB = (DAB fraction area/tissue fraction area) × 100

Values obtained for SIRT1, Nrf2, HO-1, NF-κB, and caspase-3 were used for statistical comparison between groups. All immunohistochemical analyses were performed under identical conditions to ensure reproducibility across all primary antibodies.

### 4.6. Immunofluorescence Analysis

pAkt activity was assessed using an immunofluorescence method. Formalin-fixed paraffin-embedded 5 µm-thick sections were deparaffinized and rehydrated. Antigen retrieval was performed by heating the slides in a 0.01 M citrate buffer (pH 6.0) at 95 °C for 30 min. After rising in 0.1 M PBS, nonspecific staining was blocked with a commercial protein-blocking reagent (ab236466, Abcam, Cambridge, UK) for 10 min. The sections were incubated with the primary antibody anti-phospho-Akt (4060, Cell Signaling Technology, Danvers, MA, USA) at a dilution of 1:100. This incubation was carried out overnight at 4 °C. The next day, the slides were washed in PBS before incubation with secondary anti-rabbit IgG (H + L), F(ab’)2 fragment Alexa Flour^®^ 488 Conjugate (4412, Cell Signaling Technology, Danvers, MA, USA), diluted 1:1000 for 1 h at room temperature. Nuclei were stained with 4′,6-diamidino-2-phenylindole ((DAPI), Cell Signaling Technology, 4083, Danvers, MA, USA) and imaged using a fluorescent microscope (Leica DM 6000B, Wetzlar, Germany). Fluorescence intensity was quantified using ImageJ software (version 1.54p, Wayne Rasband and contributors National Institutes of Health, Bethesda, MD, USA), and the resulting values are presented as the mean fluorescence intensity (MFI). All immunofluorescence analyses were performed under identical conditions to ensure reproducibility.

### 4.7. Statistical Analysis

Statistical analysis was conducted using IBM-SPSS software version 20.0 (SPSS, Inc., Chicago, IL, USA). Descriptive statistics, including mean, standard error, and standard deviation, were utilized. After performing the Shapiro–Wilk test for normality, suitable parametric or non-parametric tests were applied. Differences between groups were assessed using ANOVA for parametric data, while non-parametric variables were analyzed with the Kruskal–Wallis and Mann–Whitney U tests. For *post hoc* analysis, Tukey and Bonferroni tests were implemented. As this study was exploratory, no formal *a priori* power calculation was performed.

## 5. Conclusions

This study demonstrated that UDCA provides significant protection against LPS-induced myocardial injury by modulating oxidative stress, inflammation, and apoptosis, through the activation of the SIRT/Nrf2/HO-1 and inhibition of Akt/NF-κB signaling pathways, and the inhibition of pro-inflammatory and pro-apoptotic proteins.

## Data Availability

The original contributions presented in this study are included in the article/Supplementary Materials (Tables S1–S6). Further inquiries can be directed to the corresponding author.

## Supplementary Materials

The supporting information can be downloaded at: https://www.mdpi.com/article/10.3390/ijms27062843/s1.

## References

[1] Singam A. (2024). Myocardial Injury as a Harbinger of Multi-organ Failure in Septic Shock: A Comprehensive Review. Cureus.

[2] Lima M.R., Silva D. (2023). Septic cardiomyopathy: A narrative review. Rev. Port. Cardiol..

[3] Shokrizadeh H., Babaei H., Imani M., Kheirandish R. (2019). Short-and long-term effects of lipopolysaccharide-induced endotoxemia on mice ovarian tissue: Histomorphometrical evaluation. Vet. Arh..

[4] Nežić L., Škrbić R., Amidžić L., Gajanin R., Kuča K., Jaćević V. (2018). Simvastatin Protects Cardiomyocytes Against Endotoxin-induced Apoptosis and Up-regulates Survivin/NF-κB/p65 Expression. Sci. Rep..

[5] Liu G.H., Qu J., Shen X. (2008). NF-kappaB/p65 antagonizes Nrf2-ARE pathway by depriving CBP from Nrf2 and facilitating recruitment of HDAC3 to MafK. Biochim. Biophys. Acta..

[6] Minelli A., Grottelli S., Mierla A., Pinnen F., Cacciatore I., Bellezza I. (2012). Cyclo(His-Pro) exerts anti-inflammatory effects by modulating NF-κB and Nrf2 signalling. Int. J. Biochem. Cell Biol..

[7] Abu-Baih R.H., Abu-Baih D.H., Abdel-Hafez S.M.N., Fathy M. (2024). Activation of SIRT1/Nrf2/HO-1 and Beclin-1/AMPK/mTOR autophagy pathways by eprosartan ameliorates testicular dysfunction induced by testicular torsion in rats. Sci. Rep..

[8] Ding S., Liu D., Wang L., Wang G., Zhu Y. (2020). Inhibiting MicroRNA-29a Protects Myocardial Ischemia-Reperfusion Injury by Targeting SIRT1 and Suppressing Oxidative Stress and NLRP3-Mediated Pyroptosis Pathway. J. Pharmacol. Exp. Ther..

[9] Manning B.D., Toker A. (2017). AKT/PKB Signaling: Navigating the Network. Cell.

[10] O’Neill L.A., Golenbock D., Bowie A.G. (2013). The history of Toll-like receptors-redefining innate immunity. Nat. Rev. Immunol..

[11] Akira S., Takeda K. (2004). Toll-like receptor signalling. Nat. Rev. Immunol..

[12] Bai D., Ueno L., Vogt P.K. (2009). Akt-mediated regulation of NFkappaB and the essentialness of NFkappaB for the oncogenicity of PI3K and Akt. Int. J. Cancer.

[13] Luyendyk J.P., Schabbauer G.A., Tencati M., Holscher T., Pawlinski R., Mackman N. (2008). Genetic analysis of the role of the PI3K-Akt pathway in lipopolysaccharide-induced cytokine and tissue factor gene expression in monocytes/macrophages. J. Immunol..

[14] Schabbauer G., Tencati M., Pedersen B., Pawlinski R., Mackman N. (2004). PI3K-Akt pathway suppresses coagulation and inflammation in endotoxemic mice. Arterioscler. Thromb. Vasc. Biol..

[15] Zhang R., Ma W.Q., Fu M.J., Li J., Hu C.H., Chen Y., Zhou M.M., Gao Z.J., He Y.L. (2021). Overview of bile acid signaling in the cardiovascular system. World J. Clin. Cases.

[16] Li C., Yang J., Wang Y., Qi Y., Yang W., Li Y. (2020). Farnesoid X Receptor Agonists as Therapeutic Target for Cardiometabolic Diseases. Front. Pharmacol..

[17] Eblimit Z., Thevananther S., Karpen S.J., Taegtmeyer H., Moore D.D., Adorini L., Penny D.J., Desai M.S. (2018). TGR5 activation induces cytoprotective changes in the heart and improves myocardial adaptability to physiologic, inotropic, and pressure-induced stress in mice. Cardiovasc. Ther..

[18] Milivojac T., Grabež M., Krivokuća A., Maličević U., Gajić Bojić M., Đukanović Đ., Uletilović S., Mandić-Kovačević N., Cvjetković T., Barudžija M. (2024). Ursodeoxycholic and chenodeoxycholic bile acids attenuate systemic and liver inflammation induced by lipopolysaccharide in rats. Mol. Cell. Biochem..

[19] Moon I.J., Yoo H., Paik S.H., Kim H.T., Kim S.Y., Song Y., Chang S.E. (2021). Ursodeoxycholic Acid May Inhibit Environmental Aging-Associated Hyperpigmentation. Antioxidants.

[20] Hanafi N.I., Mohamed A.S., Sheikh Abdul Kadir S.H., Othman M.H.D. (2018). Overview of Bile Acids Signaling and Perspective on the Signal of Ursodeoxycholic Acid, the Most Hydrophilic Bile Acid, in the Heart. Biomolecules.

[21] Ferraro E., Pozhidaeva L., Pitcher D.S., Mansfield C., Koh J.H.B., Williamson C., Aslanidi O., Gorelik J., Ng F.S. (2020). Prolonged ursodeoxycholic acid administration reduces acute ischaemia-induced arrhythmias in adult rat hearts. Sci. Rep..

[22] Yang H., Luo F., Wei Y., Jiao Y., Qian J., Chen S., Gong Y., Tang L. (2021). TGR5 protects against cholestatic liver disease via suppressing the NF-κB pathway and activating the Nrf2/HO-1 pathway. Ann. Transl. Med..

[23] Wan Y.Y., Sheng L. (2018). Regulation of bile acid receptor activity. Liver Res..

[24] Lou Y., Shi H., Sha N., Li F., Gu X., Lin H. (2025). Ursodeoxycholic acid protects against sepsis-induced acute kidney injury by activating Nrf2/HO-1 and inhibiting NF-κB pathway. BMC Nephrol..

[25] Li X., Hu Y., He B., Li L., Tian Y., Xiao Y., Shang H., Zou Z. (2023). Design, synthesis and evaluation of ursodeoxycholic acid-cinnamic acid hybrids as potential anti-inflammatory agents by inhibiting Akt/NF-κB and MAPK signaling pathways. Eur. J. Med. Chem..

[26] Catorce M.N., Gevorkian G. (2016). LPS-induced Murine Neuroinflammation Model: Main Features and Suitability for Pre-clinical Assessment of Nutraceuticals. Curr. Neuropharmacol..

[27] Sun F., Xu K., Zhou J., Zhang W., Duan G., Lei M. (2023). Allicin protects against LPS-induced cardiomyocyte injury by activating Nrf2-HO-1 and inhibiting NLRP3 pathways. BMC Cardiovasc. Disord..

[28] Tan Y., Tu Y., Tian D., Li C., Zhong J.K., Guo Z.G. (2015). ST-elevation myocardial infarction following systemic inflammatory response syndrome. Cardiovasc. J. Afr..

[29] Tiruvoipati R., Sultana N., Lewis D. (2012). Cardiac troponin I does not independently predict mortality in critically ill patients with severe sepsis. Emerg. Med. Australas..

[30] Bao M., Liang M., Sun X., Mohyuddin S.G., Chen S., Wen J., Yong Y., Ma X., Yu Z., Ju X. (2022). Baicalin Alleviates LPS-Induced Oxidative Stress via NF-κB and Nrf2-HO1 Signaling Pathways in IPEC-J2 Cells. Front. Vet. Sci..

[31] Smith A., John M., Trout R., Davis E., Moningi S. (2009). Elevated cardiac troponins in sepsis: What do they signify?. West Va. Med. J..

[32] Futterman I.D., Jain H., McLaren R.A., Mays J.K. (2024). Cord blood troponin I levels: Biomarker evidence of fetal cardiac injury in intrahepatic cholestasis of pregnancy. AJOG Glob. Rep..

[33] Mihajlović D., Đukanović Đ., Gajić Bojić M., Jovičić S., Mandić-Kovačević N., Uletilović S., Maksimović Ž.M., Pavlović N., Dojčinović B., Bolevich S. (2024). Cardioprotective Effects of Ursodeoxycholic Acid in Isoprenaline-Induced Myocardial Injury in Rats. Biomolecules.

[34] Coelho Neto A., Azevedo R.P., Santos M.B., Galdieri L.D.C., D’Almeida V., Amaral J.L., Freitas F.G., Machado F.R. (2010). Homocysteine plasma levels as a marker of clinical severity in septic patients. Rev. Bras. Ter. Intensiv..

[35] Ploder M., Kurz K., Spittler A., Neurauter G., Roth E., Fuchs D. (2010). Early increase of plasma homocysteine in sepsis patients with poor outcome. Mol. Med..

[36] Matsunaga N., Yoshioka Y., Fukuta Y. (2021). Extremely high troponin levels induced by septic shock: A case report. J. Med. Case Rep..

[37] Ji J., Feng M., Niu X., Zhang X., Wang Y. (2021). Liraglutide blocks the proliferation, migration and phenotypic switching of Homocysteine (Hcy)-induced vascular smooth muscle cells (VSMCs) by suppressing proprotein convertase subtilisin kexin9 (PCSK9)/low-density lipoprotein receptor (LDLR). Bioengineered.

[38] Nadinskaia M., Maevskaya M., Ivashkin V., Kodzoeva K., Pirogova I., Chesnokov E., Nersesov A., Kaibullayeva J., Konysbekova A., Raissova A. (2021). Ursodeoxycholic acid as a means of preventing atherosclerosis, steatosis and liver fibrosis in patients with nonalcoholic fatty liver disease. World J. Gastroenterol..

[39] Obeidat H.M., Althunibat O.Y., Alfwuaires M.A., Aladaileh S.H., Algefare A.I., Almuqati A.F., Alasmari F., Aldal’in H.K., Alanezi A.A., Alsuwayt B. (2022). Cardioprotective Effect of Taxifolin against Isoproterenol-Induced Cardiac Injury through Decreasing Oxidative Stress, Inflammation, and Cell Death, and Activating Nrf2/HO-1 in Mice. Biomolecules.

[40] Sokolovic D., Nikolic J., Kocic G., Jevtovic-Stoimenov T., Veljkovic A., Stojanovic M., Stanojkovic Z., Sokolovic D.M., Jelic M. (2013). The effect of ursodeoxycholic acid on oxidative stress level and DNase activity in rat liver after bile duct ligation. Drug Chem. Toxicol..

[41] Monick M., Carter A.B., Robeff P.K., Flaherty D.M., Peterson M.W., Hunninghake G.W. (2001). Lipopolysaccharide activates Akt in human alveolar macrophages resulting in nuclear accumulation and transcriptional activity of beta-catenin. J. Immunol..

[42] Ryu K.Y., Lee H.J., Woo H., Kang R.J., Han K.M., Park H., Lee S.M., Lee J.-Y., Jeong Y.J., Nam H.-W. (2019). Dasatinib regulates LPS-induced microglial and astrocytic neuroinflammatory responses by inhibiting AKT/STAT3 signaling. J. Neuroinflammation.

[43] Saponaro C., Cianciulli A., Calvello R., Dragone T., Iacobazzi F., Panaro M.A. (2013). The PI3K/Akt pathway is required for LPS activation of microglial cells. Immunopharmacol. Immunotoxicol..

[44] Zhou J., Lin H., Lv T., Hao J., Zhang H., Sun S., Yang J., Chi J., Guo H. (2022). Inappropriate Activation of TLR4/NF-κB is a Cause of Heart Failure. Cardiovasc. Innov. Appl..

[45] Arisawa S., Ishida K., Kameyama N., Ueyama J., Hattori A., Tatsumi Y., Hayashi H., Yano M., Hayashi K., Katano Y. (2009). Ursodeoxycholic acid induces glutathione synthesis through activation of PI3K/Akt pathway in HepG2 cells. Biochem. Pharmacol..

[46] Paumgartner G. (2006). Medical treatment of cholestatic liver diseases: From pathobiology to pharmacological targets. World J. Gastroenterol..

[47] Mao H., Wang L., Xiong Y., Jiang G., Liu X. (2022). Fucoxanthin Attenuates Oxidative Damage by Activating the Sirt1/Nrf2/HO-1 Signaling Pathway to Protect the Kidney from Ischemia-Reperfusion Injury. Oxidative Med. Cell. Longev..

[48] Krajka-Kuźniak V., Baer-Dubowska W. (2021). Modulation of Nrf2 and NF-κB Signaling Pathways by Naturally Occurring Compounds in Relation to Cancer Prevention and Therapy. Are Combinations Better Than Single Compounds?. Int. J. Mol. Sci..

[49] Gao W., Guo L., Yang Y., Wang Y., Xia S., Gong H., Zhang B.K., Yan M. (2022). Dissecting the Crosstalk Between Nrf2 and NF-κB Response Pathways in Drug-Induced Toxicity. Front. Cell Dev. Biol..

[50] Bellezza I., Tucci A., Galli F., Grottelli S., Mierla A.L., Pilolli F., Minelli A. (2012). Inhibition of NF-κB nuclear translocation via HO-1 activation underlies α-tocopheryl succinate toxicity. J. Nutr. Biochem..

[51] Li C., Zhang S., Li L., Hu Q., Ji S. (2020). Ursodeoxycholic Acid Protects Against Arsenic Induced Hepatotoxicity by the Nrf2 Signaling Pathway. Front. Pharmacol..

[52] Ding Y.W., Zhao G.J., Li X.L., Hong G.L., Li M.F.Č., Qiu Q.M., Wu B., Lu Z.-Q. (2016). SIRT1 exerts protective effects against paraquat-induced injury in mouse type II alveolar epithelial cells by deacetylating NRF2 in vitro. Int. J. Mol. Med..

[53] Ohkawa H., Ohishi N., Yagi K. (1979). Assay for lipid peroxides in animal tissues by thiobarbituric acid reaction. Anal. Biochem..

[54] Erel O. (2005). A new automated colorimetric method for measuring total oxidant status. Clin. Biochem..

[55] Green L.C., Wagner D.A., Glogowski J., Skipper P.L., Wishnok J.S., Tannenbaum S.R. (1982). Analysis of nitrate, nitrite, and [15N]nitrate in biological fluids. Anal. Biochem..

[56] Beutler E. (1984). Red Cell Metabolism: A Manual of Biochemical Methods.

[57] Beutler E. (1982). Red Cell Metabolism. A Manual of Biochemical Methods.

[58] Beutler E., Duron O., Kelly B.M. (1963). Improved method for the determination of blood glutathione. J. Lab. Clin. Med..

[59] Bajic Z., Sobot T., Amidzic L., Vojinovic N., Jovicic S., Gajic Bojic M., Djuric D.M., Stojiljkovic M.P., Bolevich S., Skrbic R. (2024). Liraglutide Protects Cardiomyocytes against Isoprenaline-Induced Apoptosis in Experimental Takotsubo Syndrome. Biomedicines.

