# Supplementary material for: Ursodeoxycholic Acid Attenuates Lipopolysaccharide-Induced Myocardial Injury by Inhibiting Oxidative Stress, Inflammation, and Apoptosis: The Interplay of Sirt1/Nrf2 and Akt/NF-κB Signaling Pathways
Source: Int J Mol Sci. 2026 Mar 20;27(6):2843. doi: 10.3390/ijms27062843 (PMC13026358; doi:10.3390/ijms27062843)
Supplement: Supplementary file 1 [file ijms-27-02843-s001.zip › ijms-4141853-supplementary.pdf]

Table S1. Data for pAKT in Control, LPS, UDCA and LPS+UDCA groups

pAKT			
**Control**	**LPS**	**UDCA**	**LPS+UDCA**
15.006	29.417	17.442	24.136
11.493	30.223	15.627	25.377
20.566	29.257	14.001	27.990
12.404	30.009	7.448	22.126
17.075	28.922	10.572	26.246
8.913	29.482	15.291	26.231
12.495	30.220	17.051	26.123
9.826	30.240	15.228	22.842
14.216	30.390	15.385	22.310
8.289	29.520	15.545	24.653
9.982	28.775	16.133	23.705
30.000	30.218	14.234	28.545
10.298	29.154	17.098	16.224
10.278	30.123	12.325	27.247
14.861	30.739	12.966	25.769
14.358	30.323	15.524	18.654
12,717	29.505	12.453	16.065
8.052	29.532	18.528	20.866
16.416	29.693	15.090	26.917
14.511	30.909	16.853	29.004
15.577	30,434	12.392	21.247
15.674	28.742	17,489	19.512
9.959	28.538	17.686	18.015
12.605	28.383	13.699	23.898

Table S2. Data for NFκB in Control, LPS, UDCA and LPS+UDCA groups

	NFκB		
**Control**	**LPS**	**UDCA**	**LPS+UDCA**
21	30	1	4
16	42	1	2
17	34	2	2
20	35	1	8
17	40	2	7
10	61	8	5
15	40	9	4
21	43	5	4
16	43	5	3
15	52	3	5
4	20	20	8
4	24	15	12
4	27	13	12
8	25	21	21
8	29	17	35
3	22	27	26
4	29	17	28
6	37	21	19
4	26	32	21
3	28	15	18

Table S3. Data for Caspase 3 in Control, LPS, UDCA and LPS+UDCA groups

Caspase 3			
**Control**	**LPS**	**UDCA**	**LPS+UDCA**
2.0	27.0	4.0	3.0
2.0	31.0	2.0	4.0
2.0	37.0	3.0	14.0
3.00	30.00	4.00	11.00
1.00	31.00	3.00	16.00
1.00	56.00	3.00	12.00
2.00	25.00	2.00	14.00
1.00	25.00	4.00	1.00
2.00	35.00	3.00	1.00
5.00	28.00	2.00	11.00
3.00	24.00	4.00	1.00
3.00	40.00	3.00	1.00
3.00	19.00	6.00	7.00
2.00	34.00	6.00	3.00
1.00	43.00	4.00	1.00
3.00	37.00	6.00	1.00
0.00	46.00	4.00	1.00
3.00	32.00	4.00	12.00
1.00	25.00	7.00	1.00
1.00	31.00	3.00	7.00
2.05	32.80	3.85	6.10

Table S4. Data for SIRT1 in Control, LPS, UDCA and LPS+UDCA groups

SIRT1			
**Control**	**LPS**	**UDCA**	**LPS+UDCA**
43	0	11	41
20	5	29	47
27	5	16	43
42	2	52	46
24	3	45	34
51	0	45	41
53	0	46	46
74	1	48	34
67	2	37	44
58	2	55	45
30	1	20	41
22	3	27	49
65	4	10	45
29	0	27	49
25	4	24	45
51	1	13	47
46	6	21	44
47	7	26	42
57	6	27	12
72	6	17	7
43	0	11	41

Table S5. Data for Nrf2 in Control, LPS, UDCA and LPS+UDCA groups

Nrf2			
**Control**	**LPS**	**UDCA**	**LPS+UDCA**
10	8.0	29	32
7	7.0	20	36
5	12.0	31	3
16	19.0	11	11
14	3.0	25	4
8	11.0	7	45
30	2.0	26	8
31	6.0	24	13
22	13.0	33	36
12	4.0	6	5
12	3.0	28	34
12	1.0	9	21
8	0.0	25	29
17	0.0	6	28
4	0.0	18	42
23	0.0	15	23
11	0.0	8	18
19	0.0	14	17
16	1.0	29	10
19	1.0	21	22
10	8.0	29	32

Table S6. Data for HO-1 in Control, LPS, UDCA and LPS+UDCA groups

HO-1			
**Control**	**LPS**	**UDCA**	**LPS+UDCA**
56	14	44	23
43	26	45	23
60	27	62	32
52	33	40	65
38	33	36	38
29	18	18	37
62	16	41	37
55	17	38	27
20	10	46	38
48	5	36	16
45	3	49	65
49	1	51	27
44	1	62	52
44	2	42	56
49	1	53	44
62	1	59	48
28	2	61	36
27	1	43	50
40	2	49	38
38	10	56	35
56	14	44	23
